# Adult Children’s Migration and Health-Related Quality of Life Among Older Nepali Adults

**DOI:** 10.1007/s10823-024-09500-1

**Published:** 2024-02-12

**Authors:** Saruna Ghimire, Devendra Raj Singh, Sara J. McLaughlin, Dhirendra Nath, Hannah McCarren, Janardan Subedi

**Affiliations:** 1 Department of Sociology and Gerontology, Miami University, 375 Upham Hall, 100 Bishop Circle, Oxford, OH 45056 USA; 2 Scripps Gerontology Center, Miami University, Oxford, OH USA; 3 School of Human and Health Sciences, University of Huddersfield, Huddersfield, UK; 4 Southeast Asia Development Actions Network (SADAN), Lalitpur, Nepal; 5 School of Health and Allied Sciences, Pokhara University, Pokhara, Nepal

**Keywords:** Health-related quality of life, Migration, Nepal, Older adults

## Abstract

Traditionally, adult children have served as primary caretakers and providers for older Nepali adults. However, out-migration of adult children for employment and other opportunities is increasing. Health-related quality of life (HRQOL) in older Nepali adults in general and in the context of adult children’s migration is poorly understood. This study aims to assess HRQOL of older Nepali adults and its relationship with adult children’s migration. We used existing cross-sectional survey data on 260 older adults from Krishnapur municipality, which has witnessed a high rate of adult migration. HRQOL, quantified using the SF-12 scale, is expressed in terms of a physical (PCS) and mental (MCS) health component. A higher PCS and MCS score, each ranging from 0 to 100, indicates better physical and mental health, respectively. The correlates of HRQOL were assessed in simple and multiple linear regression. Participants had suboptimal HRQOL [mean (± SD): PCS = 40.4 ± 9.2 and MCS = 45.2 ± 7.7]. After adjusting for covariates, adult children’s migration was associated with lower MCS scores (β: -2.33, 95%CI: -4.21, -0.44). Individuals with more than one child had higher MCS scores (β: 2.14, 95%CI: 0.19, 4.09). Females (β: -3.64, 95%CI: -7.21, -0.06) and those with a history of unemployment (β: -6.36, 95%CI: -10.57, -2.15) had lower PCS scores than their respective counterparts. The presence of chronic conditions was associated with significantly lower PCS and MCS scores. Our findings suggest that adult children’s migration may negatively affect HRQOL among older Nepali adults, specifically their psychological well-being. Further research investigating potential moderating factors that may serve as important buffers is needed.

## Introduction

Population aging and migration are co-occurring in Nepal. The population of older Nepali adults, officially defined as those 60 years and older (Nepal Law Commission, 2006), is growing increasing from 8.14% in 2011 to 10.21% in 2021 (Central Bureau of Statistics, 2012; National Statistics Office, 2021). This growth exceeds the overall population growth rate, which decreased from 1.35% in 2011 to 0.92% in 2021 (Central Bureau of Statistics, 2012; National Statistics Office, 2021). Importantly, this pattern is expected to continue into the distant future. Another factor impacting the Nepali population is migration. Between 2007 and 2017, more than 3.5 million labor work permits were issued, enabling Nepali adults to migrate to other countries— especially those in the Middle East— for employment (Ministry of Labour and Employment, 2018). Remittance, defined as the money that migrants send back to families in origin countries, is an important source of income for migrant households and national revenue in Nepal, accounting for nearly 26% of the nation’s gross domestic product in 2016/2017 (The World Bank Group, 2018).

Scholars have acknowledged the inherent limitations of conventional health indicators such as mortality and morbidity and espoused the need for measures of quality of life (Fallowfield, 1990). Health-related quality of life (HRQOL) refers to “an individual’s or a group’s perceived physical and mental health over time” (Centers for Disease Control and Prevention, 2018). To date, only a handful of studies on HRQOL have been conducted among older Nepali adults (Dev et al., 2014; Ghimire, Baral et al., 2018), and only one previous study (Thapa et al., 2020) examined it in the context of children’s migration. Furthermore, reported estimates of HRQOL in Nepal are based on studies conducted in Kathmandu—the capital of Nepal—with outpatient (Ghimire, Baral et al., 2018) and nursing home (Dev et al., 2014) populations. Thus, they may underestimate HRQOL among community-dwelling older adults.

Institutional support for older adults is largely nonexistent in Nepal, with adult children, specifically sons and daughters-in-law, obliged to care and provide for their older parents (Geriatric Center Nepal, 2010). Given that older parents depend on their children for instrumental and emotional support, the high rate of adult children’s migration may negatively impact the overall well-being of left-behind parents. Although concern for left-behind parents has been expressed for decades (see, for example, Goldstein and Beall’s (1982) investigation of older Sherpas), scientific understanding of the impact of adult migration on health-related quality of life among older Nepali adults is underdeveloped.

With respect to HRQOL and migration, two contrasting hypotheses can be proposed. On the one hand, the financial support received from migrant children may enhance the economic condition of their parents and, thereby, improve HRQOL. On the other hand, given that children are primary caretakers of older parents in Nepal, the absence of caretakers may have adverse effects. Using the World Health Organization Quality of Life-BREF scale, Thapa et al. (2020) found a positive association between internal migration (defined as having any child currently residing in another municipality within the same province or in a different province) and the physical domain (assessed via seven items pertaining to activities of daily life) and the environmental domain (comprised of eight items related to financial resources, freedom, safety and security, health and social care, physical and home environment, and transport) among older Nepali individuals living in Arghakachi and Rupandehi districts in Nepal. However, findings for the psychological and social domains were null (Thapa et al., 2020).

While evidence on HRQOL in older Nepali adults is limited, evidence from around the globe suggests that adult children’s migration may be positively associated with depressive symptoms and loneliness in left-behind parents (Mosca & Barrett, 2016; Song, 2017; Thapa et al., 2018; Torres et al., 2018), especially mothers (Mosca & Barrett, 2016; Torres et al., 2018). A study in China, however, found no significant differences in HRQOL or chronic comorbidities (e.g., diabetes, dyslipidemia, and stroke) among older parents who were and were not left behind (Chang et al., 2016). This study aims to build the knowledge base on HRQOL in older Nepali adults, particularly as it pertains to its relationship with adult children’s migration.

## Subjects and Methods

### Study Design and Settings

Data are from a previous community-based cross-sectional study conducted for the purpose of gathering information on the significance of migration of adult children to the well-being of left-behind older parents (Ghimire, Singh et al., 2018). Data were collected between June and September 2017 in the Krishnapaur municipality of Kanchanpur district in far western Nepal.

Kanchanpur district, situated in the southwestern plains of Nepal where it borders India, has historically experienced a high migration rate. This is primarily because of its proximity to the open Indian border, which Nepalis can cross for work without permits or visas (Gill & Hoebink, 2003). In addition, a significant number of young individuals from Kanchanpur have migrated to Gulf countries and Malaysia for employment opportunities, and there is a noticeable and increasing trend in labor permit applications from the district (Ministry of Labour and Employment, 2018). Notably, one in three households in the study district has at least one household member abroad (National Statistics Office, 2021), making it an ideal location for studying migration-related challenges. For our study, we randomly selected Krishnapur municipality out of the total 20 municipalities in the Kanchanpur district. Approximately 9% of the population of Krishnapaur is aged 60 years or older (National Statistics Office, 2021).

### Sample Size and Sampling

Details on the calculation of sample size and sampling techniques are provided elsewhere (Ghimire, Singh et al., 2018). In brief, 260 individuals (approximately 10% of the older population in Krishnapaur) were selected using systematic random sampling of households. In order to participate in the study, individuals had to meet the following criteria: at least 60 years of age, resident of the study municipality for one or more years, and have an adult child (≥ 18 years of age; adopted, step, or biological).

### Data Collection and Study Variables

In-person interviews were conducted in the Nepali language by undergraduate public health students. Students were given two days of in-depth training on the purpose of the study and study procedures.

#### Dependent Variable: Health-Related Quality of Life

HRQOL was measured using the 12-Item Short-Form Health Survey (SF-12) (Ware et al., 1996).The SF-12 is comprised of 12 Likert scale items capturing perceptions of physical (PCS: Physical Component Summary) and mental (MCS: Mental Component Summary) health. Both the PCS and MCS range from 0 to 100, with higher scores indicating better physical and mental health, respectively.

#### Independent Variables

Migration of adult children (yes/no) was operationalized as having at least one child in the household who had migrated for employment. To exclude migration that did not directly generate remittance, we did not consider non-income generating migration (e.g., migration related to family conflicts or education).

Other variables included participants’ age, sex (male/female), educational status (illiterate/literate), past occupation (unemployed, housewife, agriculture, wage-based), household income, number of children, and chronic conditions. Participants’ self-reported household income was categorized into quintiles. Eight chronic conditions (diabetes, hypertension, heart disease, kidney disease, respiratory problem, gastritis, arthritis, and depression) were considered. For each of the conditions, participants were asked if a health provider had ever diagnosed the condition. The number of chronic conditions was summed and categorized as none, one condition, and two or more conditions.

### Statistical Methods

Histograms and the Kolmogorov-Smirnov test were used to assess the distribution of continuous variables. Descriptive statistics (frequencies, percentages, means, or medians, as applicable) were calculated for all study variables. Mean differences in PCS and MCS scores by participants’ characteristics were assessed using independent sample t-tests or ANOVA, as applicable. Simple and multiple linear regression were used to examine correlates of PCS and MCS. Variables with a p-value ≤ 0.1 in unadjusted models were included in multiple linear regression. These included: age, sex, education, past occupation, number of children, number of chronic conditions, and adult children’s migration. Multicollinearity was not a problem, as variance inflation factors were between 1 and 2.

## Results

### Participants’ Characteristics

Table 1 provides the descriptive findings by HRQOL scale. The median age of participants was 67 years, and the majority of participants were male (57.7%), illiterate (69.6%), involved in agriculture in the past (36.9%), and had more than one chronic condition (41.2%). Just over half of participants had at least one migrant child (Table 1).

**Table 1:** Participants’ characteristics by SF-12 scales

	Overall (*N* = 260)	PCS	*p*-value^a^	MCS	*p*-value^a^
	N (%) or Median (IQR)	Mean ± SD		Mean ± SD	
**Age, years, median (IQR)**	67 (63–72)				
**Age categories**					
60–69 years	148 (56.9)	41.5 ± 8.5		46.2 ± 7.7	
≥ 70 years	112 (43.1)	38.8 ± 10.0	**0.019**	44.0 ± 7.6	**0.026**
**Sex**					
Male	150 (57.7)	42.1 ± 9.0	**< 0.001**	46.6 ± 8.1	**0.001**
Female	110 (42.3)	38.0 ± 9.1		43.4 ± 6.9	
**Educational status**					
Illiterate	181 (69.6)	40.1 ± 9.0	0.431	44.4 ± 7.7	**0.005**
Literate	79 (30.4)	41.1 ± 9.8		47.2 ± 7.6	
**Past occupation**					
Unemployed	23 (8.8)	37.7 ± 7.2	**< 0.001** ^**§**^	45.2 ± 9.2	**< 0.001** ^**§**^
Housewife	82 (31.5)	37.3 ± 8.7		42.4 ± 6.3	
Agriculture	96 (36.9)	41.3 ± 9.5		46.4 ± 7.5	
Wage-based	59 (22.7)	44.1 ± 8.8		47.4 ± 8.3	
**Household income (USD), median (IQR)** ^**1**^	83.3 (50.0-133.3)				
**Quintiles of household income**					
1	51 (19.6)	39.5 ± 8.5	0.541	44.2 ± 8.5	0.372
2	44 (16.9)	39.2 ± 9.6		46.0 ± 6.4	
3	51 (19.6)	39.8 ± 9.4		44.2 ± 8.0	
4	59 (22.7)	41.1 ± 9.4		45.1 ± 7.7	
5	55 (21.2)	41.8 ± 9.3		46.7 ± 7.9	
**Number of children**					
One	132 (50.8)	39.8 ± 9.4	0.323	44.1 ± 8.1	**0.017**
More than one	128 (49.2)	40.9 ± 9.1		46.4 ± 7.2	
**Chronic conditions**					
None	59 (22.7)	46.0 ± 8.2	**< 0.001** ^**§§**^	48.6 ± 7.3	**< 0.001** ^**§§**^
One	94 (36.2)	40.8 ± 8.8		45.6 ± 7.5	
Two or more	107 (41.2)	36.9 ± 8.6		43.0 ± 7.5	
**Migration of adult children**					
Yes	133 (51.2)	40.5 ± 8.6	0.753	44.3 ± 8.0	0.056
No	127 (48.8)	40.2 ± 9.9		46.2 ± 7.4	

^a^P-values from an independent sample t-test or ANOVA

^§^Post-hoc pairwise comparisons showed that the mean PCS and MCS scores for housewife were significantly different from agriculture and wage-based employment (*P* < 0.05)

^§§^Post-hoc pairwise comparisons showed that the PCS and MCS means were significantly different across the numbers of chronic conditions (*P* < 0.05)

^1^One US dollar = 100 Nepali rupee

*Abbreviation* PCS - Physical Health Composite Scores, MCS - Mental Health Composite Scores

The mean (± SD) PCS and MCS scores were 40.4 ± 9.2 and 45.2 ± 7.7, respectively. In bivariate analyses, older participants (≥ 70 years), females, and those with one or more chronic conditions had significantly lower PCS and MCS scores (Table 1). Additionally, the MCS score was higher among literate participants and those who had more than one child (Table 1).

### Correlates of HRQOL

After adjusting for covariates, adult children’s migration was associated with a significantly lower MCS score. Specifically, on average, older adults who had children who had migrated for income-related purposes had an MCS score that was 2.33 units lower than their counterparts who did not (β: -2.33, 95%CI: -4.21, -0.44). No significant association was observed for adult children’s migration and PCS.

In adjusted analyses, females (β: -3.64, 95%CI: -7.21, -0.06) and those who were unemployed in the past (β: -6.36, 95%CI: -10.57, -2.15) had lower PCS scores than their respective counterparts. In addition, compared to participants who had no chronic conditions, those with one (β: -4.48, 95%CI: -7.29, -1.67) or more (β: -7.70, 95%CI: -10.53, -4.87) chronic conditions had significantly lower PCS scores (Table 2).

**Table 2:** Simple and multiple linear regression for correlates of HRQOL: Physical and Mental Health Composite Scores

	Physical health β (95%CI)		Mental health β (95%CI)	
	Unadjusted	Adjusted^1^	Unadjusted	Adjusted^1^
**Adult children’s migration (Ref**: No**)**				
Yes	0.36 (-1.90, 2.63)	0.95 (-1.23, 3.14)	-1.84 (-3.72, 0.05)	**-2.33 (-4.21, -0.44)**
**Age categories (Ref**: 60–69 years**)**				
≥ 70 years	**-2.69 (-4.95, -0.43)**	-1.99 (-4.15, 0.17)	**-2.15 (-4.05, -0.26)**	-1.06 (-2.92, 0.81)
**Sex (Ref**: Male**)**				
Female	**-4.07 (-6.31, -1.84)**	**-3.64 (-7.21, -0.06)**	**-3.16 (-5.04, -1.28)**	-0.18 (-3.31, 2.95)
**Educational status (Ref**: Literate**)**				
Illiterate	-0.98 (-3.44, 1.47)	2.33 (-0.30, 4.96)	**-2.88 (-4.91, -0.85)**	-1.27 (-3.54, 1.00)
**Past Occupation (Ref**: Wage-based**)**				
Unemployed	**-6.39 (-10.70, -2.08)**	**-6.36 (-10.57, -2.15)**	-2.21 (-5.85, 1.43)	-0.52 (-4.15, 3.12)
Housewife	**-6.76 (-9.75, -3.76)**	-2.59 (-6.79, 1.61)	**-5.05 (-7.58, -2.52)**	-3.18 (-6.81, 0.45)
Agriculture	-2.72 (-5.62, 0.18)	-2.90 (-5.82, 0.02)	-1.02 (-3.46, 1.43)	-0.61 (-3.13, 1.91)
^**2**^**Quintiles of household income (Ref**: 1**)**				
2	-0.38 (-4.13, 3.37)	-	1.77 (-1.37, 4.91)	-
3	0.21 (-3.40, 3.82)	-	-0.01 (-3.03, 3.01)	-
4	1.60 (-1.89, 5.09)	-	0.91 (-2.01, 3.82)	-
5	2.26 (-1.29, 5.80)	-	2.52 (-0.44, 5.48)	-
**Number of children (Ref**: One**)**				
Two or more	1.14 (-1.12, 3.40)	0.48 (-1.78, 2.73)	**2.29 (0.41, 4.16)**	**2.14 (0.19, 4.09)**
**Chronic conditions (Ref**: None**)**				
One	**-5.26 (-8.07, -2.45)**	**-4.48 (-7.29, -1.67)**	**-3.04 (-5.48, -0.60)**	**-2.58 (-5.00, -0.15)**
Two or more	**-9.11 (-11.85, -6.37)**	**-7.70 (-10.53, -4.87)**	**-5.60 (-7.98, -3.22)**	**-4.28 (-6.72, -1.83)**

^1^Model adjusted for age, sex, education, past occupation, number of children, number of chronic conditions, and adult children’s migration

^2^Household income was not included in adjusted models because it was not significant at *p* < 0.10 in the unadjusted analysis. Statistically significant beta coefficients and 95% CIs are bolded

Having more than one child increased the MCS score by 2.14 units (β: 2.14, 95%CI: 0.19, 4.09) in adjusted analyses. Number of chronic conditions was negatively associated with MCS; participants with one (β: -2.58, 95%CI: -5.00, -0.15) or more (β: -4.28, 95%CI: -6.72, -1.83) chronic conditions had lower MCS scores. Although age was inversely associated with both PCS and MCS in unadjusted analyses, statistical significance was lost once covariates were controlled (Table 2).

## Discussion

We examined the association of adult children’s migration and HRQOL in older Nepali adults. While no significant association was evident for physical HRQOL, we observed a significant association between adult children’s migration and mental HRQOL. Specifically, older Nepali adults who reported having an adult child migrate for income-related purposes rated their quality of life in the mental health domain lower than those who did not.

Existing literature on the well-being of left-behind parents is inconsistent. While some studies have found no association between adult children’s migration and the mental health of left-behind older parents (Bohme et al., 2015; Gibson et al., 2011; Thapa et al., 2020), our findings are consistent with several studies reporting a significant adverse relationship (Antman, 2010; Mosca & Barrett, 2016; Song, 2017; Torres et al., 2018). In addition, an integrative review of 25 studies, mostly cross-sectional, concluded that older adults with a migrant child experience poor mental health outcomes such as depression and loneliness (Thapa et al., 2018).

While we did not find a significant association with the physical domain, less favorable physical health was observed in Mexico among left-behind parents relative to parents living with their children (Antman, 2010). In contrast, in a previous study of older adults in Nepal, children’s internal migration was associated with more favorable quality of life in the physical domain (Thapa et al., 2020). Differences in sample composition, the instrument used to measure QOL, the type of migration examined, and derived benefits of migration may explain the observed differences.

The disparate findings for physical and mental HRQOL observed in this study may partly reflect the long-standing role of family caregiving. Specifically, in Nepal’s joint/extended family system, children reside together with their older parents. It is, therefore, likely that even if an adult child has migrated, the older parent continues to receive care and support from other children and family members. While this care may serve to reduce physical ailments, the older person may nonetheless experience a sense of sadness over the lack of direct contact with their migrated child. Concern about the health and safety of their migrant child may also be a factor, as there are widespread reports of work-related accidents and injuries as well as abuse, fraud, and economic exploitation among Nepali migrant workers (Adhikary et al., 2019; Aryal et al., 2019; Joshi et al., 2011; Ministry of Labour and Employment, 2018). Qualitative work with older Nepali adults on the impact of having a migrant child would enhance understanding of these findings as well as the supports needed to promote optimal mental health. Notably, mental health is stigmatized in Nepal and is understood in terms of “madness.” Such a stereotype discourages people from opening up about their illness and/or seeking health care which is itself limited in terms of availability (Devkota, 2011).

Consistent with existing literature on older Nepali adults (Dev et al., 2014; Ghimire, Baral et al., 2018; Ghimire, Pradhananga et al., 2017), we observed suboptimal HRQOL in our sample of older adults overall. Biological senescence (e.g., reduced immunity and musculoskeletal strength) heightens older adults’ risk of physical health problems (e.g., functional difficulties and chronic disease (Cruz-Jentoft et al., 2010; Kirkwood, 2008). Besides the increasing burden of individual chronic conditions, multimorbidity is emerging as a prevalent issue among older Nepali adults (Balakrishnan et al., 2022) and is another important factor that appears to significantly impact HRQOL in older ages (Makovski et al., 2019; Williams & Egede, 2016). In our study, significantly lower physical and mental health scores were observed in those with two or more chronic conditions.

Among the demographic covariates assessed, females and those unemployed in the past had lower physical health, which has been observed in studies involving samples outside of Nepal (Cherepanov et al., 2010; Georgopoulou et al., 2011; Park et al., 2018). In a patriarchal society like Nepal, lower HRQOL among females is not surprising owing to restricted opportunities for women in multiple domains of life (health and nutrition, education, employment) (Ghimire, Baral et al., 2017; Shakya, 2014). Likewise, given the strong association between SES and health and well-being (Marmot, 2002), our finding that those with a history of unemployment reported lower HRQOL is not unexpected. Employment shapes access to resources such as income and health care that influence HRQOL at an older age.

## Limitations of the Study

This investigation in not without limitations. First, the cross-sectional study design inhibits causal inference. Future studies have the opportunity to determine the causal impact of adult children’s migration on HRQOL using longitudinal designs. Second, the small sample size may have limited our ability to detect group differences. Third, chronic conditions were measured by self-report and may not reflect actual health status. Self-reported chronic conditions are, however, widely used in population-based surveys (He et al., 2012), especially when resources are scarce. Fourth, we were unable to control for several potentially important covariates, including the duration of migration and the availability of other social support. Fifth, several aspects of the study limit generalizability. Given the urban-rural disparity in HRQOL, our findings may not be generalizable to older Nepali adults residing in rural areas. Additionally, interviews were conducted in the Nepali language. This could have resulted in the exclusion of individuals who are not fluent in Nepali and/or misinterpretation of survey items, introducing selection bias and measurement error. It is worth noting, however, that Nepali is the official language of the country, and census data indicate that 91% of the population either speaks or understands Nepali (44.86% as their mother tongue and 46.23% as a second language) (National Statistics Office, 2021). Consequently, while there may some bias, we believe it is likely minimal. In addition, given the male-to-female sex ratio in the study district (89.8; (National Statistics Office, 2021), a representative sample should include a higher number of females than males. This is not the case for our sample. This likely stems from the sampling strategy used for households with two or more age-eligible individuals. In such cases, the oldest person in the household—who is likely to be disproportionately male owing to age differences in martial relationships—was selected. Another limitation pertains to the measurement of migration. More specifically, our measure of migration does not capture its complexity. Migration may, for example, be permanent or temporary. Given our focus on labor-related migration, which is likely to be temporary, we may have underestimated the effect of migration on parent’s quality of life. Other characteristics of the migration experience that may influence observed associations, include destination country, perceived benefits of adult child migration, and the gender of the migrated child. Factors such as these may influence an older parent’s feelings about the migration of their adult child and result in qualitatively different experiences for older parents who remain at home. To gain a more comprehensive understanding of the impact of migration on the well-being of left-behind parents, future studies should consider a more nuanced assessment of adult child migration. Finally, we did not examine potential moderating factors. Future studies should investigate the extent to which cohabitating children and the wider social networks of older adults may serve as important buffers.

With these limitations noted, this study adds to the paucity of knowledge on HRQOL in the context of adult children’s migration in Nepal. HRQOL has been used as one of the benchmarks to evaluate health objectives set in national programs (e.g., Healthy People 2000, 2010, and 2020 in the US; Centers for Disease Control and Prevention, 2018). However, this concept is underemphasized in Nepal. In the absence of large nationwide studies on aging, our study supplements the two previous studies conducted in the capital city of Kathmandu (Dev et al., 2014; Ghimire, Baral et al., 2018),  providing baseline information on the HRQOL of older Nepali adults. Our study also addresses a previous review conducted for the Government of Nepal that emphasized a lack of studies on HRQOL of older Nepali adults and recommended research to fill the gaps in knowledge (Geriatric Center Nepal, 2010).

## Conclusion

Migration and population aging are two concurrent issues in Nepal. We found suboptimal HRQOL in our sample of older Nepali adults and that lower mental HRQOL is associated with adult children’s migration. Research investigating the mechanisms by which adult children’s migration may exert its effect as well as potential modifying factors is needed to determine how to minimize the mental health effects of out-migration on older adults. The influence of cohabitating children and wider social networks are potentially promising places to start.

## Data Availability

The data used in this research are available upon reasonable request. Interested parties may contact the corresponding author for access to the dataset and related information.
